# Effects of blood flow restriction training on bone turnover markers, microstructure, and biomechanics in rats

**DOI:** 10.3389/fendo.2023.1194364

**Published:** 2023-08-21

**Authors:** Yawei Song, Hao Wang, Liang Chen, Yuwen Shangguan, Hu Jia

**Affiliations:** ^1^ Jiangsu Province "Sports and Health Project" Collaborative Innovation Center, Nanjing Sport Institute, Nanjing, China; ^2^ Department of Sports and Health Sciences, Nanjing, China; ^3^ Medical school, Shandong Xiehe University, Jinan, China

**Keywords:** blood flow restriction training, bone turnover marker, bone biomechanics, microstructure, mechanics of materials

## Abstract

**Objective:**

The present study aimed to investigate the effects of blood flow restriction training on muscle strength, bone tissue structure material, and biomechanical properties in rats applying various exercise interventions and to analyze the process by identifying the bone turnover markers, it provides a theoretical basis for the application of BFRT in clinical rehabilitation.

**Methods:**

A total of 24, 3-month-old male SD (Sprague Dawley) rats were randomly divided into pressurized control group (CON, n=6), low-intensity training group (LIRT, n=6), high-intensity training group (HIRT, n=6), and blood flow restriction training group (LIBFR, n=6) for 8-week ladder-climbing exercises. The pressured control group were given only ischemia treatments and did not undertake any burden. The low-intensity training group was allowed to climb the ladder with 30% of the maximum voluntary carrying capacity (MVCC). The rats in the high-intensity training group were allowed to climb the ladder with 70% MVCC. The blood flow restriction training group climbed the ladder with 30% MVCC while imposing blood flow restriction. Before sampling, the final MVCC was measured using a ladder-climbing protocol with progressively increasing weight loading. The serum, muscle, and bone were removed for sampling. The concentrations of the bone turnover markers PINP, BGP, and CTX in the serum were measured using ELISA. The bone mineral density and microstructure of femur bones were measured using micro-CT. Three-point bending and torsion tests were performed by a universal testing machine to measure the material mechanics and structural mechanics indexes of the femur bone.

**Results:**

The results of maximum strength test showed that the MVCC in LIRT, HIRT, and LIBFR groups was significantly greater than in the CON group, while the MVCC in the HIRT group was significantly higher than that in the LIRT group (P<0.05). According to the results of the bone turnover marker test, the concentrations of bone formation indexes PINP (amino-terminal extension peptide of type I procollagen) and BGP (bone gla protein) were significantly lower in the CON group than in the HIRT group (P<0.01), while those were significantly higher in the LIRT group compared to the HIRT group (P<0.01). In terms of bone resorption indexes, significant differences were identified only between the HIRT and other groups (P<0.05). The micro-CT examination revealed that the HIRT group had significantly greater bone density index values than the CON and LIRT groups (P<0.05). The results of three-point bending and torsion test by the universal material testing machine showed that the elastic modulus and maximum load indexes of the HIRT group were significantly smaller than those of the LIBFR group (P<0.05). The fracture load indexes in the HIRT group were significantly smaller than in the LIBFR group (P<0.05).

**Conclusion:**

1. LIRT, HIRT, LIBFR, and CON all have significant differences, and this training helps to improve maximum strength, with HIRT being the most effective. 2. Blood flow restriction training can improve the expression of bone turnover markers, such as PINP and BGP, which promote bone tissue formation. 3. Blood flow restriction training can improve muscle strength and increase the positive development of bone turnover markers, thereby improving bone biomechanical properties such as bone elastic modulus and maximum load.

## 1 Introduction

The age of the elderly population has gradually increased in the 21^st^ century. From 2000, the elderly population has reached 88.11 million, accounting for about 6.96% of the total population of China. Until 2010, the elderly population had exceeded 160 million people, accounting for 12% of the total population (1). The increase in the rate of aging population indicates that China has begun to enter the most rapid stage of aging development (2). Over the past two decades, studies on the causes, diagnosis, and treatment of osteoporosis in the elderly have attracted widespread attention and become a central focus of gerontological medical study. Statistically, about 2 million people in the >20-years-old age group suffer from osteoporosis in China (3). However, due to age and disease factors, it is difficult for this group to enhance muscle strength and bone tissue intensity through intense training, which affects the effectiveness of rehabilitation. Thus, blood flow restriction training is developed as an emerging technique (4), mainly through mechanical compression to block the blood flow to the limb. The blockage of venous return also reduces the inflow of arterial blood to the distal end, causing a large volume of blood to collect in the pressurized area, causing hypoxia. This technique is gaining significant attention in the field of sports training and clinical medicine. Compared to traditional strength training, blood flow restriction training requires low load to induce muscle hypertrophy, increase muscle strength, reduce mechanical stress on joints and bones, and provide an alternative solution for people who are not suitable for high-load training but need to increase muscle strength (5). In past studies, blood flow restriction training has been proven to be effective, but it is not well known and initially causes discomfort (6). Blood flow restriction induces hypoxia and metabolic effects, as well as reduced protein hydrolysis and induction of synthetic metabolic processes. Current research confirms that increased strength in men and women is usually associated with muscle hypertrophy (7). After measuring the elevation of bone markers, there are preliminary reports on the positive effects on bone health. According to reports, after BFR training, the strength of non occlusive muscles also increased. BFRT can improve strength and reduce atrophy of the knee extensor during fixation (8). There have been controversial observations regarding changes in aerobic capacity. But so far, there is no standard BFR training guide.

Hitherto, studies on blood flow restriction training have focused on the effects on muscle strength, muscle hypertrophy, and the cardiovascular circulatory system, with less focus on the effects on the bone. The present study supported the hypothesis that blood flow restriction training might provide a novel approach to induce muscle and bone adaptations and increase bone tissue formation. In summary, this study aimed to investigate the effects of blood flow restriction training on bone tissue through animal experiments. Thus, blood flow restriction training animal model established to compare the control and different intensity groups to explore the changes in muscle, bone turnover markers, bone structure morphology, and bone intensity indicators.

## 2 Materials and methods

### 2.1 Animal experiments

Three-month-old male SD (Sprague Dawley) rats weighing about 240 g were randomly divided into four groups (six rats per group). These rats were provided with the National standard food for rodents and water ad libitum. The temperature of the animal rearing room was 22 ± 2°C, and the relative humidity (RH) was 30–45%.

### 2.2 Animal grouping

Three-month-old rats were purchased and acclimatized for one week. After weighing and numbering, the animals were randomly divided into a pressurized control group (CON, n=6), low-intensity training group (LIRT, n=6), high-intensity training group (HIRT, n=6), and blood flow restriction training group (LIBFR, n=6).

### 2.3 Training scheme

#### 2.3.1 Ladder-climbing training protocol

**Figure 1:**
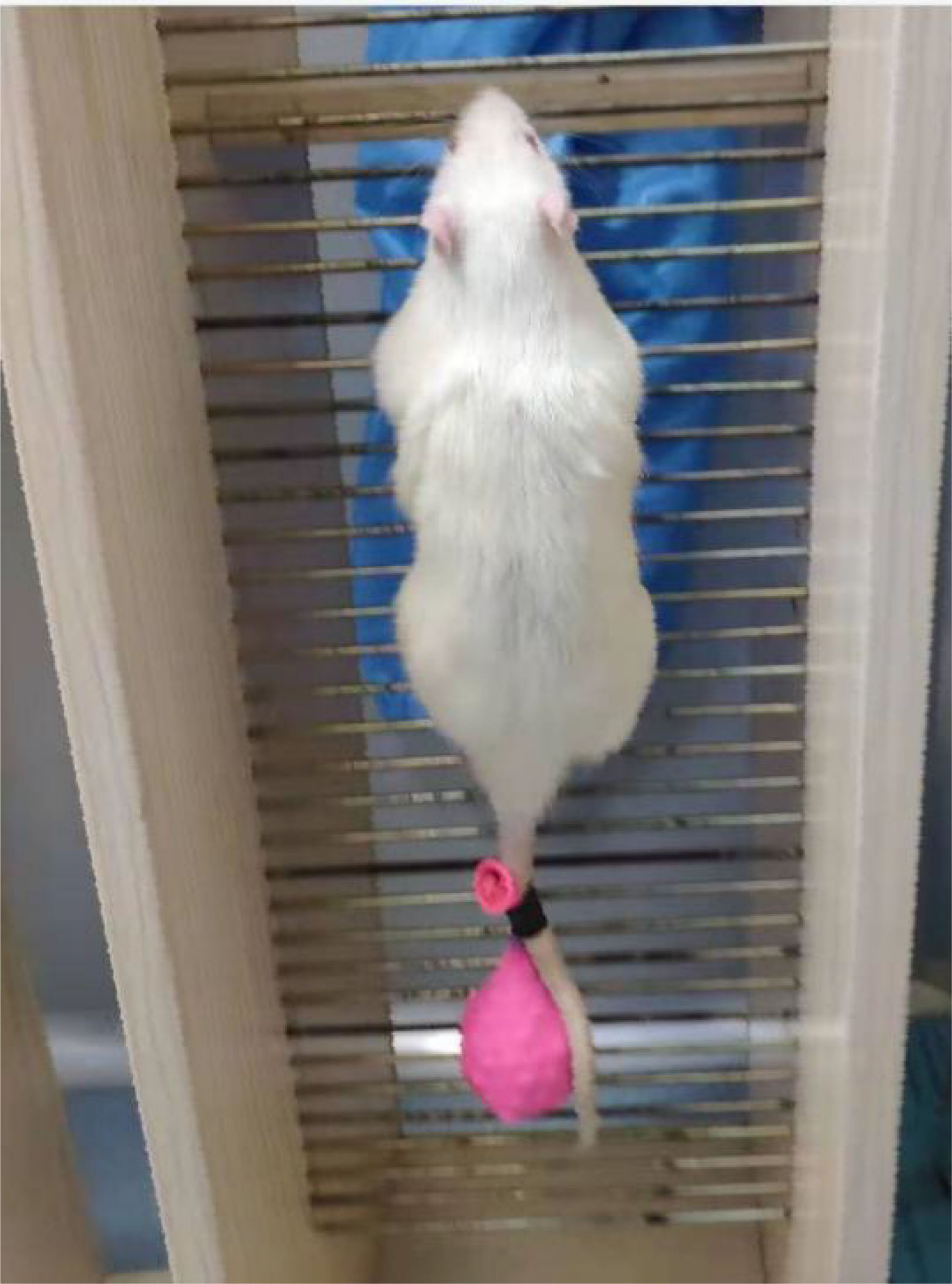
Weighted-ladder climbing training. The tail of the rat is subjected to a load and is stimulated to quickly climb to the top.

This is shown in **Figure 1**, rats used ladder climbing for resistance training, and the resistance training method was tail weight-bearing ladder climbing. The rats climbed a 1-m ladder with a 0.5-cm grid inclining at 85°. The ladder contained 54 steps (2), with a platform at the top of these steps, which could accommodate up to two rats resting simultaneously. The rats with weights attached to their tails were trained to climb the ladder with appropriate strength.

#### 2.3.2 Blood flow restriction training

**Figure 2:**
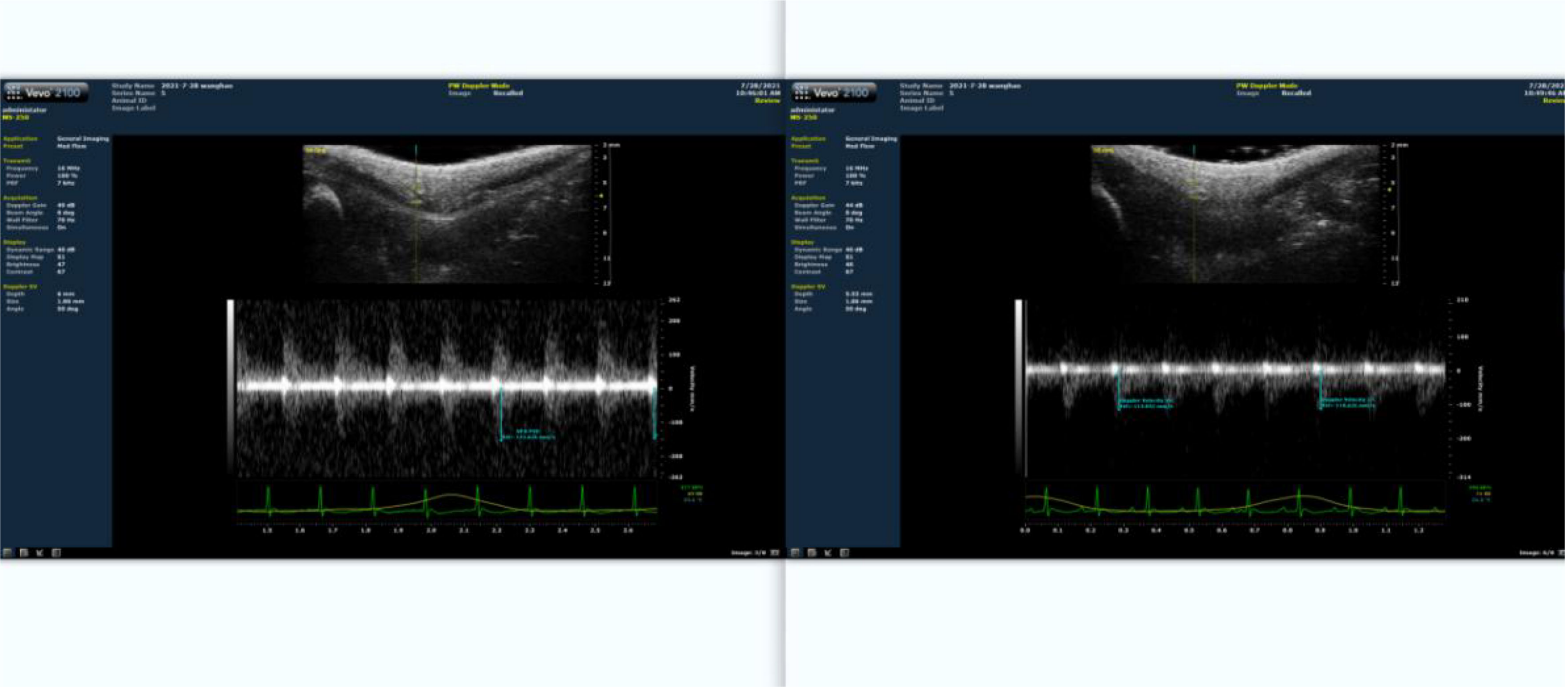
Proportion of blood flow restriction detected by ultrasound. The left panel depicts the situation prior to blood flow restriction. The right panel represents the results of blood flow limitation.

The rat femoral artery at the root of the thigh in the lower limb was probed using high-frequency ultrasound biomicroscopy (**Figure 2**). The blood flow velocity at this time point was recorded. Then bind a rubber band about 2mm below the groin of the rat, and measure the blood flow velocity at this moment. Adjust the length of the rubber band to ensure that the blood flow is limited by about 30-40%, and the optimal length is then obtained. Ultrasonography was conducted every three weeks to adjust the length of the rubber band during training. Control the rats to complete a single ladder climbing training within 8-10 s, and only apply pressure treatment during this training period.

#### 2.3.3 Maximum voluntary carrying capacity test

This is shown in **Table 1**. The load was placed in a thickened balloon and attached to their tails through electrical tape. The initial attached weight was 25% of the animals’ body weight. When the rats reached the platform of the ladder, they were allowed to rest for 5 min. The tests were conducted in the order of 25%, 50%, 75%, and 100% load. After completing the ladder climbing with 100% of their body weight, the subsequent load was increased by 30 g until the rats could not complete the test. The previous load was the maximum voluntary carrying capacity (MVCC) of the tested rats (9).

**Table 1:** Ladder-climbing training protocol.

Group	Interventions	Training times	Intergroup interval
Ischemic control group	Blood flow restriction	15	1 min
Low-intensity training group	30% MVCC load	15	1 min
High-intensity training group	70% MVCC load	15	1 min
Ischemic training group	30% blood flow restriction+30% MVCC load	15	1 min

#### 2.3.4 Sample collection

**Figure 3:**
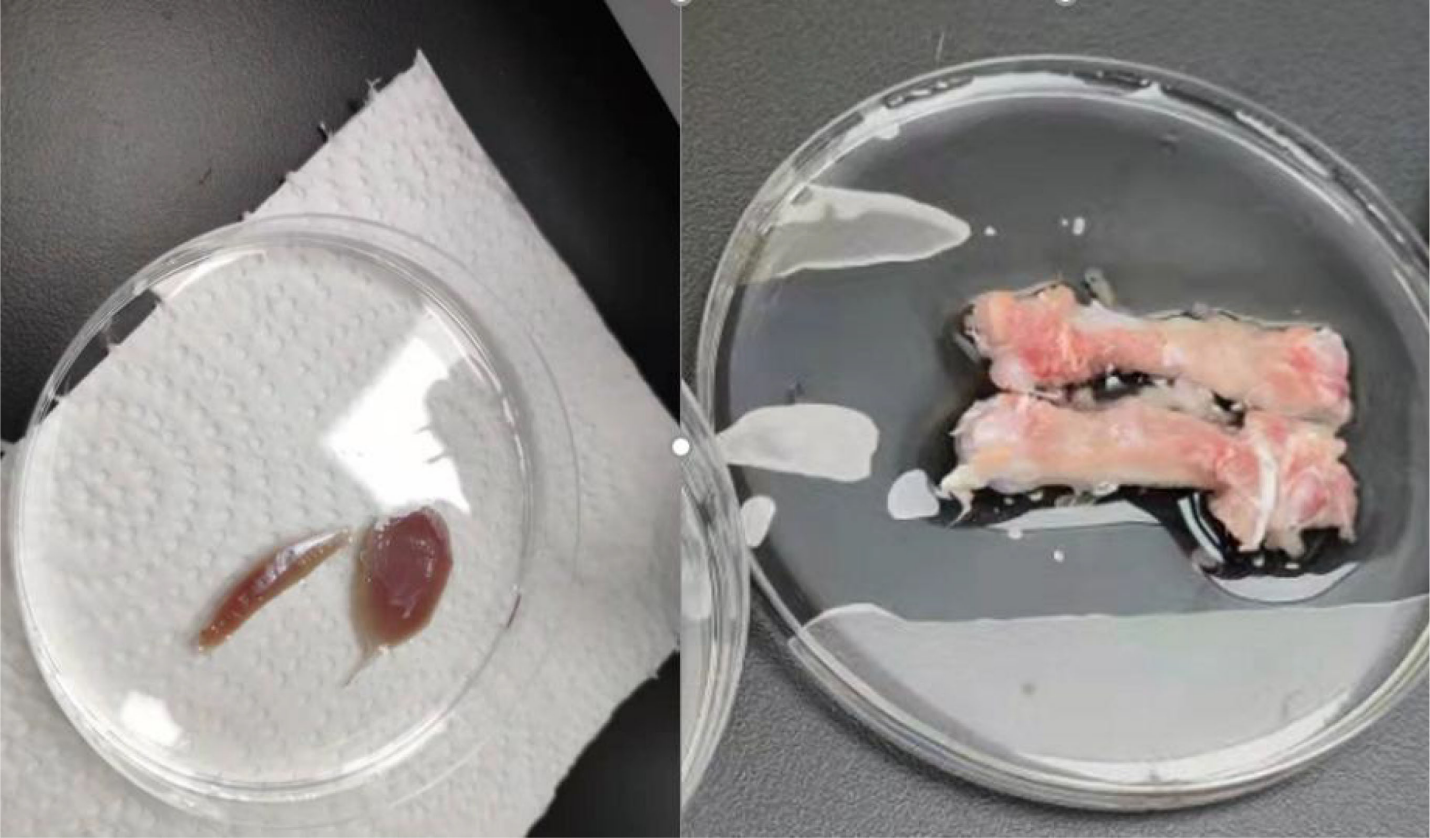
Animal tissue sampling. In the left image, the left muscle is the long toe extensor. The right is the tibialis anterior muscle.In the right image, It’s the bilateral femurs of rats that have been peeled off. The instrument used is high-frequency color ultrasound for small animals, with the model being Vevo 2110.

The serum, muscle tissue, and bone samples were prepared for analysis. After the rats were anesthetized, blood was collected from the abdominal aorta, incubated at room temperature for 2 h, and the upper layer of serum was extracted by centrifugation at 3000 rpm for 15 min. The lower limbs were divided bilaterally. Firstly, locate the tibialis anterior extensor muscle and toe extensor muscle of the rat, separate them along the muscle belly towards both ends, find the starting and ending points of the tendons at both ends, and cut them for later use, weighed,the removed muscles and bones are shown in **Figure 3**, and stored at −80°C in EP tubes. After removing the surface tissue, the femur was placed at −20°C.

### 2.4 Methods for identifying indicators

#### 2.4.1 Detection of rat serum-related indexes

Enzyme-linked immunosorbent assay (ELISA) was used to detect bone turnover markers in rats. The serum was extracted, and the concentrations of BGP (osteocalcin), PINP (amino-terminal extension peptide of type I procollagen), and β-CTX (beta-collagen degradation product) were measured by the corresponding ELISA kits (Jianglan Pure Biological Reagent Co., LTD, Jiangxi, China.).

The biochemical indicators in this study are explained as follows:

##### 2.4.1.1 BGP

The specific binding of hydroxyapatite promotes the formation of hydroxyapatite crystals, thereby increasing bone salt content and improving bone strength, which is often used as a specific index reflecting osteogenic activity (10).

##### 2.4.1.2 β-CTX

It was negatively correlated with the level of type I collagen, and the breakdown of type I collagen showed enhanced bone resorption, which could reflect the degree of bone resorption (11).

##### 2.4.1.3 PINP

The level of expression of PINP reflects the formation of new bone (12). PINP is derived from type I procollagen. When osteoblastic synthesis decreases, PINP levels, which reflect changes in newly synthesized type I collagen, drop (13).

#### 2.4.2 Detection of bone density and bone microstructure indexes in rats

The isolated bones stored at −20°C were thawed at room temperature for several hours. Microfocus-computed tomography was performed on the samples using a micro-CT system (SkyScan1176, Brooke, Inc, Germany.) with a spatial resolution of 18 μm. The main parameters of the selected 3D microstructure included trabecular thickness (Tb.Th), trabecular number (Tb.N), trabecular separation/spacing (Tb.Sp), and bone volume fraction (BV/TV).

#### 2.4.3 Detection of biomechanical indexes of rat bones

##### 2.4.3.1 Three-point bending test

**Figure 4:**
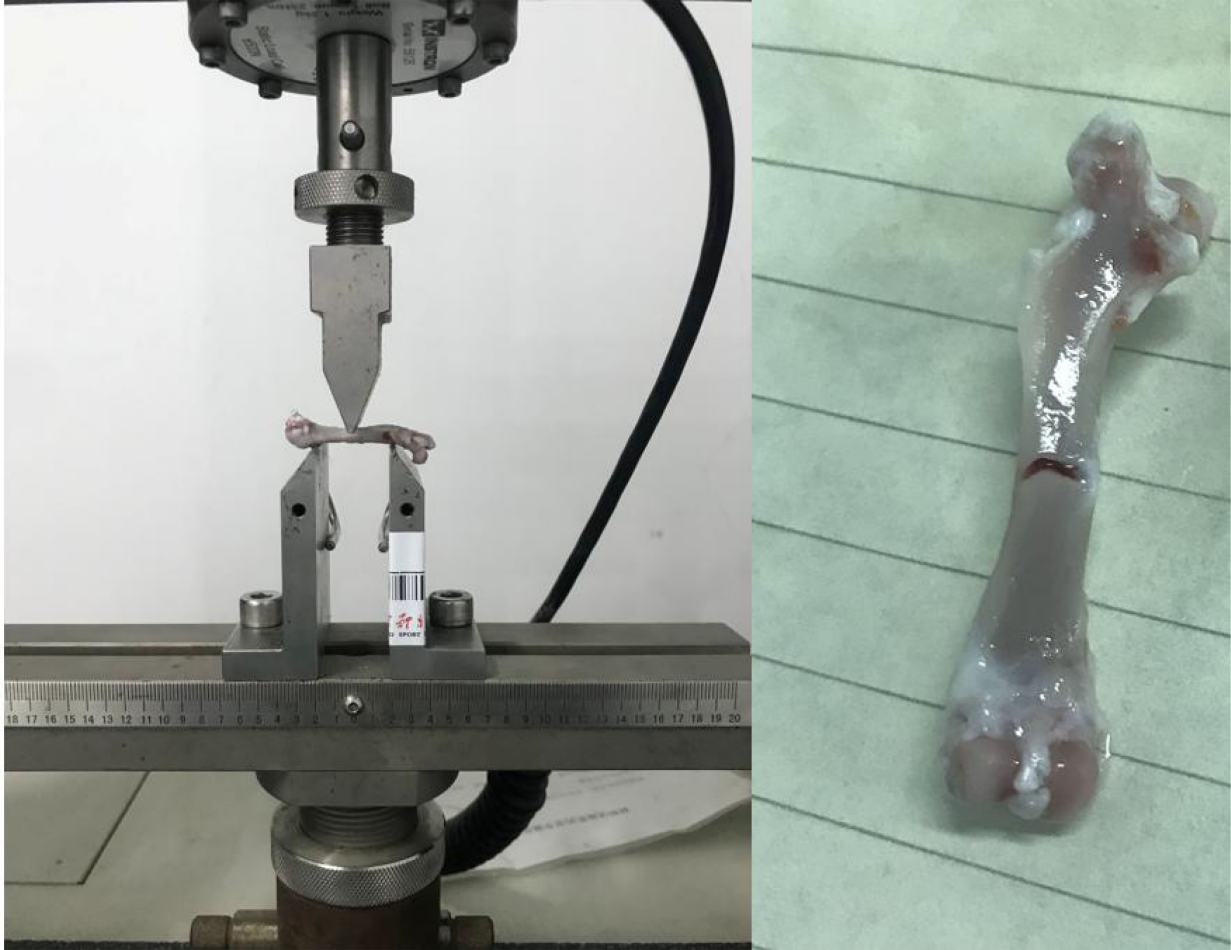
Schematic of the three-point bending experiment. The left image shows the initial state of the three-point bending test, with the femur placed on a bracket to begin the test. The right image shows the femur that fractured after the bending test. The instrument used is a universal material testing machine from Instron Company in the United States, model 3367.

**Figure 5:**
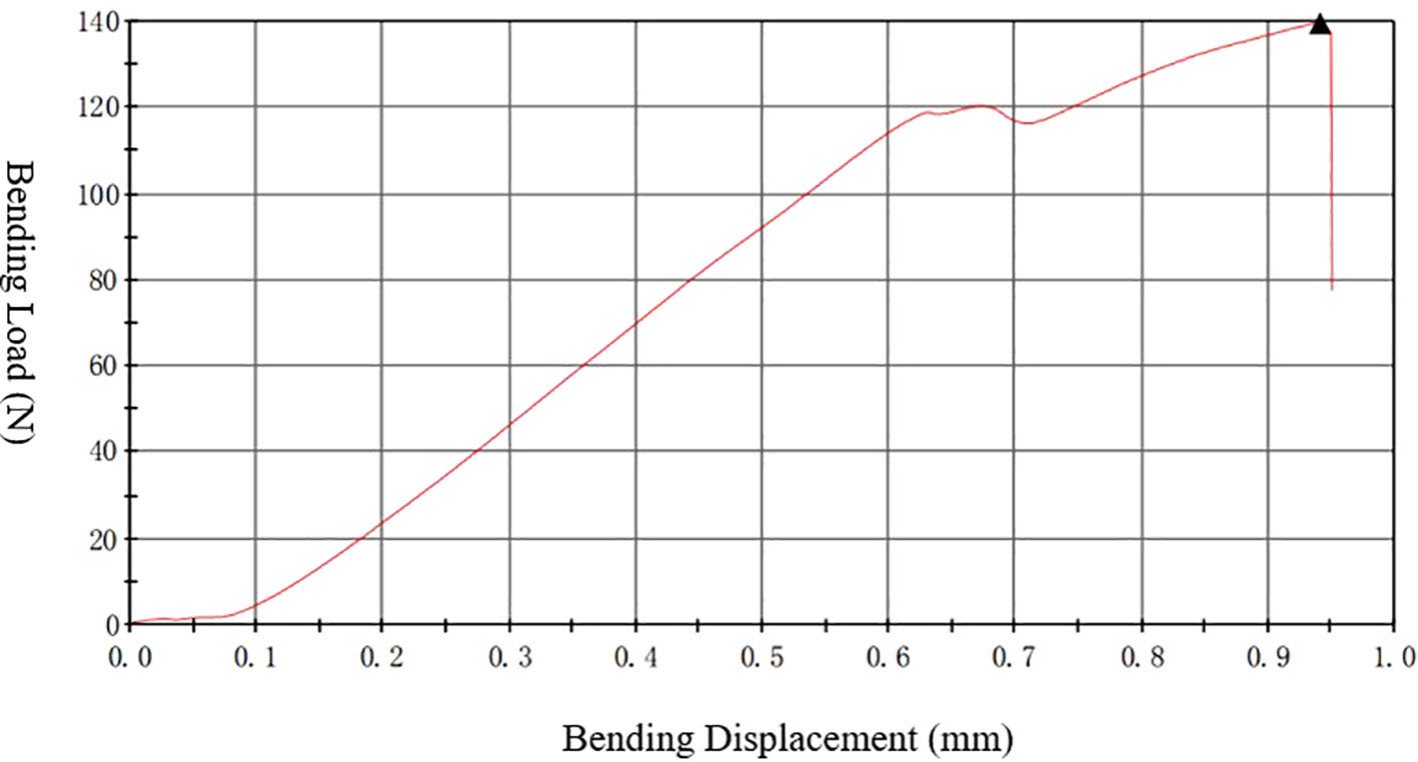
Three-point bending “force-displacement” curve. The instrument used is a universal material testing machine from Instron Company in the United States, model 3367.

The left femur was mounted on the surface, with the concave side facing upward. This is shown in **Figure 4**, both sides were placed above the support fixture, adjusting the span distance between the support jig to 20 mm. The loading rate was 1 mm/min, and the stopping condition was set to cease when the load was dropped to 40%; consequently, the femur fractured is shown in **Figure 4**. The maximum load, fracture load, and maximum deflection were calculated according to the force-displacement curve,the change curve is shown in **Figure 5**.

In this study, the femur cross-section at the broken end was modeled as an elliptical circle. The cross-sectional moment of inertia (I) and the bending modulus of elasticity (Ec) was calculated using the following formulas.


(Formula 1) $$\text{I}=\frac{(BH^{3}-bh^{3})\pi }{64}$$

(Formula 2) $${\text{E}}_{c}=\frac{L^{3}}{48I}(\frac{\Delta F}{\Delta f})$$

Ec is the sample elastic modulus, △F is the increment of bending force, △f is the corresponding increment of deflection, the ratio of △F and △f is the slope of the force-displacement curve, and L is the distance between the support fixtures.

##### 2.4.3.2 Experimental approach for torsional mechanics

**Figure 6:**
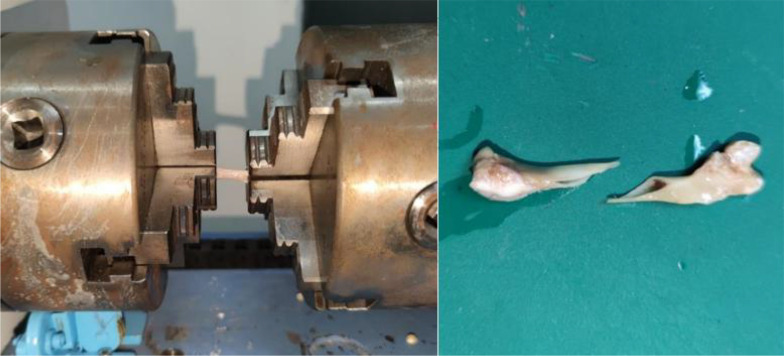
Schematic of torsional mechanics experiment. The left image shows the initial state of the torsion test, with the femur placed on a bracket to begin the test. The right image shows the femur that fractured after the torsion test. The instrument used is a universal material testing machine from Instron Company in the United States, model 8850.

**Figure 7:**
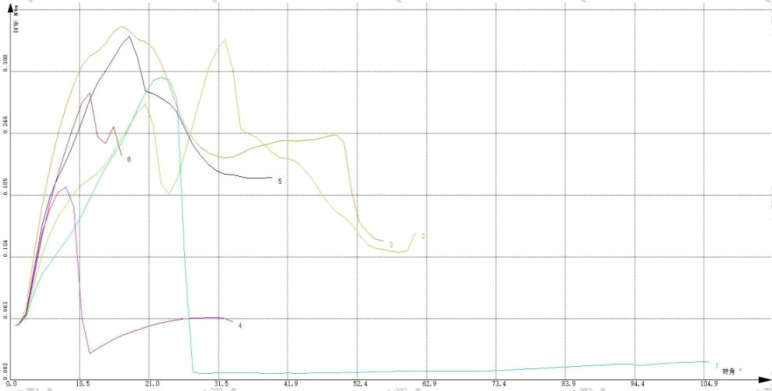
“Force-displacement” curve of torsion test. The instrument used is a universal material testing machine from Instron Company in the United States, model 8850.

The right femur of the rat was selected, and the test environment was 29.1°C/42% RH. The samples were placed in the fixture of the torsion material testing machine with a speed of 3 rpm. The experiment ended when the material fragmented. The test was stopped when the sample fractured,the bone placement and post-fracture samples are shown in **Figure 6**. The torsion angle at the maximum torque was determined from the reading of the material testing machine at the time of fracture. The maximum torque was calculated based on the force-displacement curve, this Force-displacement curve is shown in **Figure 7**.

### 2.5 Statistical analysis

Parameters were expressed as mean ± standard deviation (SD). All statistical analyses of the data were conducted using SPSS 25.0. The data were analyzed by one-way analysis of variance (ANOVA), while both pre- and post-experiment parameters were analyzed using two-way ANOVA. Data were further analyzed using the least significant difference (LSD) *post-hoc* test. P<0.05 was considered statistically significant, and P<0.01 indicated a highly significant level.

## 3 Results

### 3.1 MVCC and muscle weight of rats in each group

The maximum voluntary load-bearing capacity of different groups of rats is shown in **Table 2**. Before training, there was no significant difference in the maximum voluntary load-bearing capacity between each group of rats (P>0.05). After 8 weeks of training, the MVCC of each group improved. Intergroup control showed that the MVCC of the LIRT group, HIRT group, and LIBFR group was higher than that of the CON group (P<0.01). In addition, the MVCC of the HIRT group was higher than that of the LIRT group (P<0.05), and there was no significant difference between the other groups (P>0.05).

**Table 2:** Changes in MVCC of rats.

Variables	CON	LIRT	HIRT	LIBFR
Initial MVCC(g)	280.67 ± 50.687	264 ± 44.33	257.83 ± 33.71	269.67 ± 40.53
final MVCC(g)	652.16 ± 24.70	1040.33 ± 85.33^**^	1148.5 ± 53.24^#**^	1103.83 ± 141.18^**^

Compared with the CON group, **P<0.01, and compared with the LIRT group, ^#^P<0.05.

The values of anterior skeletal muscle/body weight and extensor digitorum longus/body weight in the LIRT group, HIRT group, and LIBFR group are shown in **Table 3**, with significant differences compared to the CON group (P<0.05). The HIRT group has the highest mean, followed by the LIBFR group, but there is no significant difference (P>0.05)

**Table 3:** Muscle weight of rats in each group.

Variables	CON	LIRT	HIRT	LIBFR
tibialis anterior/weight	0.00175 ± 0.00017	0.00205 ± 0.00012**	0.00205 ± 0.00011**	0.00200 ± 0.00016**
extensor digitorum longus/weight	0.00044 ± 0.00004	0.00048 ± 0.00002**	0.00050 ± 0.00001**	0.00049 ± 0.00001**

Compared with the CON group, **P<0.01.

### 3.2 Bone turnover markers of rats in each group

After 8 weeks of training, the results of the bone turnover markers in each group of rats are shown in **Table 4**. In the bone formation index, the PINP and BGP values of the CON group were significantly lower than those in the HIRT and LIBFR groups (P<0.01). The PINP and BGP values of the LIRT group were significantly lower than those in the HIRT and LIBFR groups (P<0.01). Moreover, the HIRT group was significantly lower than the other groups in terms of bone resorption index (P<0.05).

**Table 4:** Concentration of bone turnover markers in rats.

Variables	CON	LIRT	HIRT	LIBFR	F	η²	
PINP (pg/mL)	337.55 ± 6.85	330.21 ± 12.65	376.68 ± 8.24^**##^	368.53 ± 9.03^**##^	35.02	0.84
BGP (ng/mL)	3.80 ± 0.15	3.63 ± 0.22	4.12 ± 0.10^**##^	4.05 ± 0.11^**##^	13.64	0.67
β-CTX (pg/mL)	29.42 ± 1.98	28.14 ± 2.87	26.20 ± 2.20^*^	27.67 ± 2.83	18.53	0.71

*P<0.05, **P<0.01 compared to the CON group and ^##^P<0.01 compared to the LIRT group. The F values are the ANOVA statistic and the η² is the effect size of the ANOVA analysis.

### 3.3 Bone mineral density (BMD) and skeletal microstructure of rats in each group

As shown in **Table 5**, only the BMD indexes of the HIRT group were significantly higher than those of the CON and LIRT groups in this study (P<0.05). The remaining indexes showed no significant differences.

**Table 5:** Parameters of micro-CT scan in rats.

Variables	CON	LIRT	HIRT	LIBFR	F	η²
BMD (g/cm³)	0.79 ± 0.04^*^	0.79 ± 0.02^*^	0.83 ± 0.02	0.81 ± 0.02	3.40	0.34
BV/TV (m)	30.11 ± 3.29	30.33 ± 4.46	32.00 ± 6.55	31.94 ± 7.37	0.19	0.03
Tb.Th (1/mm)	0.10 ± 0.00	0.10 ± 0.00	0.10 ± 0.01	0.10 ± 0.01	0.32	0.05
Tb.N (m)	3.14 ± 0.31	3.18 ± 0.35	3.25 ± 0.33	3.18 ± 0.56	0.04	0.00
Tb.Sp (%)	0.23 ± 0.03	0.23 ± 0.02	0.21 ± 0.04	0.22 ± 0.033	0.59	0.08

*P<0.05 compared to the HIRT group. The F values are the ANOVA statistic and the η² is the effect size of the ANOVA analysis.

### 3.4 Biomechanical properties of rats in each group

After 8 weeks of training, the bone material mechanics and bone structure mechanics measured by the three-point bending experiment and torsion test are shown in **Table 6**.

**Table 6:** Biomechanical test data.

Variables	CON	LIRT	HIRT	LIBFR	F	η²	
Elastic modulus (MPa)	1103.24 ± 112.58	1217.40 ± 109.01	1020.08 ± 108.39	1184.06 ± 167.54^*^	1.94	0.27
Maximum load (N)	135.61 ± 12.36	137.76 ± 13.17	125.85 ± 5.09	140.34 ± 14.12^*^	1.06	0.17
Fracture load (N)	78.02 ± 6.03	80.66 ± 7.20^*^	73.15 ± 2.84	81.08 ± 6.03^*^	1.53	0.22
Bending Strength (MPa)	49.69 ± 4.55	50.50 ± 4.83	46.30 ± 2.92	51.18 ± 5.40	0.73	0.12
Energy absorption (J)	0.09 ± 0.01	0.09 ± 0.03	0.09 ± 0.01	0.08 ± 0.02	0.13	0.22
Maximum Torque (Nm)	0.30 ± 0.06	0.33 ± 0.08	0.31 ± 0.06	0.26 ± 0.06	0.48	0.15
Torsion angle at maximum torque (Nm)	17.62 ± 8.48	17.22 ± 11.86	12.42 ± 6.66	8.41 ± 6.66	1.02	0.24

*P<0.05 compared to the HIRT group. The F values are the ANOVA statistic and the η² is the effect size of the ANOVA analysis.

The HIRT group was lower than the LIBFR group in terms of elastic modulus and maximum load index (P<0.05) and lower than the LIRT and LIBFR groups with respect to fracture load index (P<0.05).

## 4 Discussion

### 4.1 Effect of blood flow restriction training on bone microarchitecture

#### 4.1.1 Mechanisms underlying the influence of blood flow restriction training on bone microarchitecture

The results of this study showed that the LIBFR group was significantly higher than the CON and LIRT groups in terms of bone density index (P>0.05), but the LIBFR group was significantly higher than the LIRT and CON groups with respect to bone turnover index (P<0.05). This finding suggested that blood flow restriction training was effective in bone tissue creation but could not completely represent the bone microstructure. Similar results have been illustrated previously (2).

Currently, there is no clear explanation as to the mechanism underlying the phenomenon that blood flow restriction can promote positive bone tissue development. Presently, the most accepted explanation is that blood flow restriction results from increased interstitial fluid flow throughout the bone tissue due to high venous occlusion and consequently increased intramedullary pressure gradient (14). The mechanical forces acting on the bone during exercise are generated by muscle contraction, impact forces from contact with the ground, or a combination of both. These forces cause strain at the tissue level. Therefore, mechanical loads are crucial for the reconstruction of bone. Typically, osteocytes sense strain mainly through changes in the intraosseous fluid flow. The external load stimulation creates pressure gradients within the bone traps (3), which affect the intraosseous fluid flow changes. During load-induced strain, the pressure gradient drives the interstitial fluid flow (IFF) from the deformation zone to the tension zone, increasing the fluid shear stress in the osteocyte membrane/cellular processes, which in turn results in biochemical responses. Due to the difficulty in directly measuring the interstitial fluid flow *in vivo* through the bone trap-vertebral canal system, *in vivo* measurement of intramedullary pressure (IMP) has been employed to describe intraosseous pressure and fluid environment in bone. Qin et al. (4) implanted pressure transducers in the marrow cavity of weight-bearing bone in the animals to investigate the effects of loading, IMP, and bone adaptation. The study also observed a significant increase in bone volume and endosteal and periosteal new bone formation in the middle ulna exposed to fluid loading stimuli, which could be attributed to the anabolic stimulation by IMP and increased fluid flow. In conclusion, these studies indirectly demonstrated the importance of pressure gradients and mechanical loading-mediated increases in the interstitial fluid flow for bone adaptation.

Bone has an abundance of vascular tissue that promotes the perfusion of oxygenated blood, removes metabolites, and provides nutrients needed by the bone to continuously form new bone and resorb the damaged bone (15). The structural and material properties are modified to ensure that strength can meet functional requirements (16, 17).

Another potential mechanism of the blood flow restriction exercise affecting bone health is through the activation of the hypoxia-inducible transcription factor (HIF) pathway and its downstream activation of vascular endothelial growth factor (VEGF) (18). Accumulating evidence suggested the presence of capillaries in the structural units of bone (19). The secretion of substances that inhibit osteoclast activity and increase osteoblast activity indicated the involvement of vascular endothelial in the coupled process of bone resorption and bone formation (20). Several pieces of evidence support the role of angiogenesis-osteogenesis coupling in the process of bone formation and repair. Although the application of blood flow restriction during exercise primarily blocks venous blood flow (21), it may also partially restrict the arterial inflow, ultimately leading to a state of acute hypoxia in the tissues below the cuff. Recent studies have shown that hypoxic pressure/hypoxia is an effective stimulus for the activation of the HIF pathway in osteoblasts, increasing the expression of VEGF and subsequently, the formation of microvessels and repair of damaged bone tissue after fracture. In animal studies, inhibition of VEGF1 and VEGF2 receptors affects bone deposition, vessel number, and healing time, leading to decreased bone formation and angiogenesis (22). Wan et al. demonstrated that BFR activates HIF, which in turn increases the expression of VEGF and microvessel formation in the bone tissue (23).

#### 4.1.2 Effects of muscle tissue on bone microarchitecture

In another pathway of adaptive changes in bone tissue, we found that muscle and bone are closely linked through anatomical features, mechanical forces, metabolism, and other function (24, 25). From an anatomical point of view (26, 27), the muscles are closely connected to bones through tendons, forming a lever system. In terms of mechanotransduction, muscle contraction forces, in addition to gravitational load, can also drive the movement of the skeleton, providing stress stimuli to the bone (28). Regarding metabolism, the endocrine-paracrine secretion between the muscle and bone can release secretory factors capable of regulating the proximal tissues and organs (29). Some studies have confirmed that strength training significantly improves lower limb muscle strength, which has a positive effect on BMD in young women and has become a predictor of the parameter. Lam et al. found that muscle electrical stimulation promotes the contraction of muscle tissue by applying muscle electrical stimulation in people lacking exercise capacity, inhibits bone resorption, and reduces the degradation of bone structure and bone material, demonstrating that muscle action can affect bone tissue.

### 4.2 Effect of blood flow restriction training on bone intensity

The current study indicated that high-intensity training increases bone strength, which is mainly expressed as bone elastic modulus and loading in bone mechanics. The elastic modulus is the intrinsic stiffness of the bone tissue, which is related to its composition, irrespective of the size and shape. Sundh et al (30). applied high-impact loads to experimental subjects. At the end of the experiment, bone microstructure, geometry, and density did not produce significant differences, while bone strength was enhanced. However, our study exhibited different results. A three-point bending test on the femur revealed that the HIRT group had overall poor results in terms of material mechanical properties and structural mechanical properties. After 8 weeks of high-intensity training, the overall strength of the femur in the HIRT group decreased. Relevant studies have shown similar findings to this experiment. Polisel et al. (31). observed that high-intensity training causes a decline in bone tissue strength in mice; similar studies have been conducted in athletes (32). In gymnasts, prolonged high-intensity training caused their overall BMD to be lower than that of the general population. High-intensity exercise leads to loss of bone mineral content (33), which reduces the mechanical properties of bone and increases the risk factor for bone injury during exercise.

Several studies have indicated that the bone tissue mineral content and bone strength decrease when the load is increased to a certain level during jumping intervention in 6-week-old rats, suggesting microdamage to the bone tissue. In addition, skeletal muscle can buffer a portion of the impact and pressure. In the examination of the muscle tissue after high-intensity training, we found that high-intensity exercise causes apoptosis, microstructural damage to skeletal muscle cells, and increases muscle fatigue (34). The reduced muscle contraction force has difficulty in countering the stresses exerted on the bone by large external forces, resulting in an uneven distribution of stresses on the bone. The bone tissue in certain areas is exposed to high levels of stress, which increases the strains and strain rates, in turn causing microdamage to the bone tissue.

## 5 Conclusions

1. LIRT, HIRT, LIBFR, and CON all have significant differences, and this training helps to improve maximum strength, with HIRT being the most effective.
2. Blood flow restriction training improves the expression of bone turnover markers, such as PINP and BGP, which promote bone tissue formation.
3. Blood flow restriction training can improve muscle strength and increase the positive development of bone turnover markers, thereby improving bone biomechanical properties such as bone elastic modulus and maximum load.

## 6 Limitation

During the experiment, we also had many overlooked details, which we need to pay attention to in future experiments. For example, when controlling the training load of rats, we only consider the weight change of the load, but ignore the volume change caused by the weight change, which may cause errors in rat training.

In this study, our research on the application of BFTR to bones only stays at the basic research stage, lacking exploration of clinical trials. In the following experiments, we will add clinical trials on the basis of basic research to better explore the effectiveness of BFTR in practical applications.

## Data availability statement

The original contributions presented in the study are included in the article/supplementary material. Further inquiries can be directed to the corresponding author.

## Ethics statement

The animal study was reviewed and approved by Nanjing Sport Institute.

## Author contributions

YWeiS, HW, and LC jointly designed the project and revised the manuscript; HW, LC, YWenS, and HJ collected and analyzed the data together; YWeiS and HW wrote the manuscript. All authors contributed to the article and approved the submitted version.
